# Effect of common foods as supplements for the mycelium growth of *Ganoderma lucidum* and *Pleurotus ostreatus* on solid substrates

**DOI:** 10.1371/journal.pone.0260170

**Published:** 2021-11-30

**Authors:** Eugene Soh, Nazanin Saeidi, Alireza Javadian, Dirk E. Hebel, Hortense Le Ferrand

**Affiliations:** 1 School of Mechanical and Aerospace Engineering, Nanyang Technological University, Singapore, Singapore; 2 School of Architecture, Karlsruhe Institute of Technology, Karlsruhe, Germany; 3 Future Cities Laboratory, Singapore ETH Centre, Singapore, Singapore; 4 School of Materials Science and Engineering, Nanyang Technological University, Singapore, Singapore; ICAR-Directorate of Mushroom Research, INDIA

## Abstract

The transition from a linear to a circular economy is urgently needed to mitigate environmental impacts and loss of biodiversity. Among the many potential solutions, the development of entirely natural-based materials derived from waste is promising. One such material is mycelium-bound composites obtained from the growth of fungi onto solid lignocellulosic substrates, which find applications such as insulating foams, textiles, packaging, etc. During growth, the fungus degrades and digests the substrate to create a web-like stiff network called mycelium. The development of the mycelium is influenced by several factors, including the substrate composition. As food waste accounts for nearly 44% of total municipal solid waste, incorporating food in the substrate composition could be a means to increase the nutrients absorbed by the fungus. In this paper, we study the effects of the addition of food supplements on the growth of two fungal species, *Ganoderma lucidum* and *Pleurotus ostreatus*. The substrates, the food supplements, and the mycelia are characterized using Fourier-transform infrared spectroscopy, scanning electron microscopy, and optical microscopy. Our results show that addition of barley as a supplement significantly boosts the growth of *G*. *lucidum* and *P*. *ostreatus*. Using a common food as a nutritious enrichment for the development of mycelium is a simple and straightforward strategy to create waste-based mycelium-bound biocomposites for a large range of applications, on-site, therefore promoting a circular economy.

## 1. Introduction

In the era of the Anthropocene, transitioning from a linear to a circular economy is a must, in order to mitigate environmental impacts and the loss of biodiversity [1, 2]. Among the various solutions to support this transition, mycelium-bound composites, which are composite materials obtained from lignocellulosic substrates and fungi, have appeared highly promising for a large variety of applications such as packaging, insulation and design [3]. Fungi can grow on a large variety of substrates, most of which can be derived from agronomic and agricultural waste. On the one hand, growing fungi for mushroom consumption, *i*.*e*. the fruiting bodies of fungi, permits the transformation of low-quality waste to high-quality food [4]. On the other hand, growing fungi for composite fabrication, *i*.*e*. using the vegetative part of the fungus called the mycelium, allows the transformation of low-quality abundant waste to affordable and recyclable products such as insulation foams, protective panels, architectural bricks, and packaging [5, 6]. Mycelium-based products can therefore support the transition to a circular economy through establishing bio-based loops [7]. Mycelium products enable the valorization of agricultural and agronomic waste, the reduction in transportation costs by being grown and fabricated on-site, and the recycling of its constituents, among other advantages. The first crucial step in this life cycle is the selection of adequate substrates for the growth of the fungus. Although the development of mycelium biocomposites has been investigated using various substrates, the effects of foods as supplements for the fungal growth and the resulting properties of the composites have not been studied extensively in the literature.

**Fig 1:**
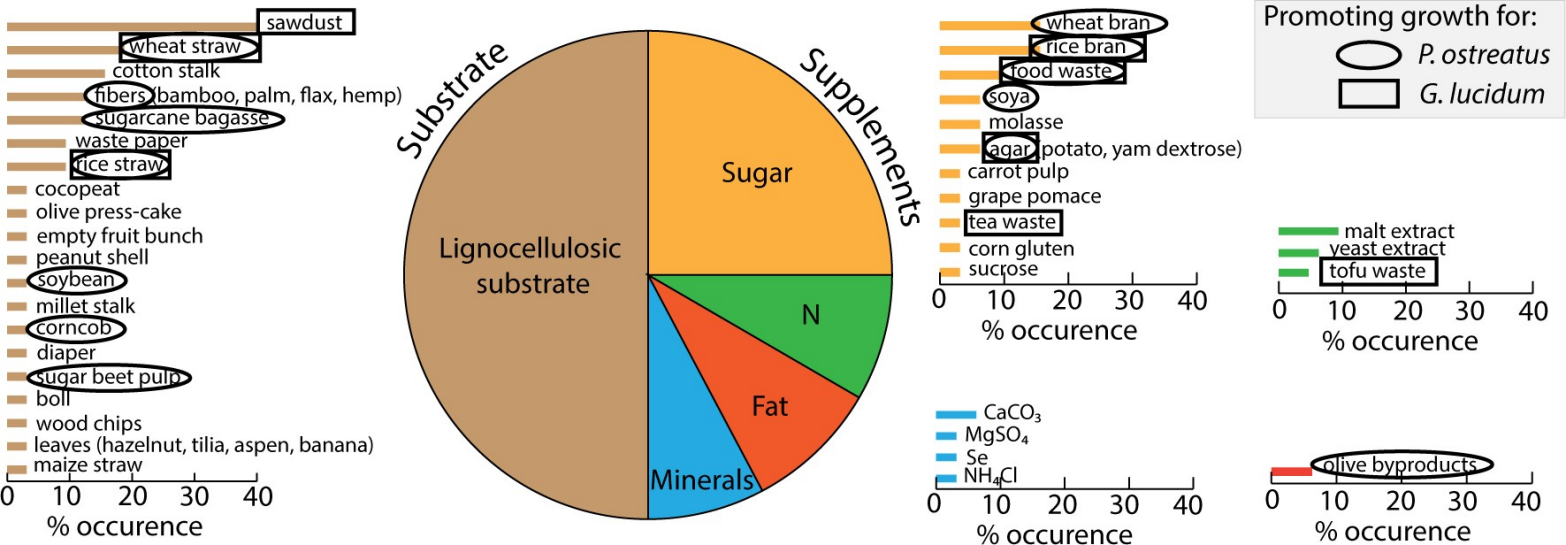
Review of substrates and supplements used for *P*. *ostreatus* and *G*. *lucidum*. The percentage of occurrence refers to the occurrence of these ingredients reported in the literature (see **Table 1** for the references). The circles and squares indicate compositions that have been reported to increase the yield of *P*. *ostreatus* and *G*. *lucidum*, respectively. The sawdust commonly used is typically from oak, alder, beech, acacia or poplar.

The most common fungal species in mycelium-based products are *Ganoderma lucidum* and *Pleurotus ostreatus*. These fungi are used extensively due to the medicinal and nutritious properties of their fruiting bodies [8, 9]. During cultivation on solid lignocellulosic substrates, the fungi obtain their nutrients by degrading the lignin of the substrates using enzymes. These nutrients are then digested and used for biosynthesis by the fungus. The composition of the substrate is therefore crucial for the development of the fungus. In nature, fungi grow on a large variety of rich organic substrates that result from decaying plants, fruits, and animals. Numerous studies of mycelium growth and mycelium-based products report the use of different types of substrates. Although the growth parameters and quality of fungi depends on many factors including temperature, humidity, light, and air flow, several substrates and supplements have been reported as preferable for the growth of the *G*. *lucidum* and *P*. *ostreatus*. The various substrates and supplements explored in the literature are summarized in **Fig 1** and **Table 1**, where the occurrence reports the percentage of research papers published to date. Among all the substrates and supplements, the ones that are circled and squared are those reported as boosting the growth of *P*. *ostreatus* and *G*. *lucidum*, respectively. As seen in both **Fig 1** and **Table 1**, many supplements resulting from agronomic waste appear to be promoting growth. However, the specific characteristics that the supplement should have in order to enhance the fungal mycelium growth and properties are not always known due to the lack of detailed characterisation of the supplements and the variation of many components available in the substrate composition.

**Table 1:** Literature overview of the influence of substrates and supplements on the growth of *G*. *lucidum* and *P*. *ostreatus*.

Fungus	Substrate	Supplements	Results	Ref
*G*. *lucidum*	Oak sawdust	Rice bran, food waste compost	15% food waste compost gave highest yield of fruiting bodies. Higher concentration reduced the yield.	[10]
*G*. *lucidum*	Sawdust of hornbeam	Tea waste, wheat bran and CaCO_3_	20% of tea waste led to highest yield. Increased growth due to high amounts of K, N, Fe, Mg.	[11]
*G*. *lucidum &*	Wheat straw	Olive by-products	Increase in the protein content of *P*. *ostreatus* mycelium and in glucan for *G*. *lucidum*.	[12]
*P*. *ostreatus*				
*G*. *lucidum*	Cotton stalk, maize straw, rice straw, sugarcane bagasse, wheat straw	Wheat bran and corn gluten	Rice straw showed the highest yield with increase in protein and polysaccharide content.	[13]
*G*. *lucidum*	Shredded cassava	Wheat bran, rice bran, MgSO_4_	Wheat bran more favourable than rice bran.	[14]
*Ganoderma &*	Spent mushroom substrate	Wheat bran and soybean flour	Food supplement increased the laccase activity and the formation of fruiting bodies.	[15]
*P*. *ostreatus*				
*G*. *lucidum*	Alder, beech and oak sawdust, flax shives	N.A.	Higher yield on alder and beech sawdust.	[16]
*G*. *lucidum*	Sawdust from acacia	Soy residue from was tofu manufacturing.	Highest rate of mycelial growth for media ratios of C to N content on the growing substrate.	[17]
*Ganoderma &*	Leaves from hazelnut, tilia, European aspen, wheat straw, beech sawdust, waste paper	Wheat bran	Wheat straw led to higher growth. Wheat bran led to contamination.	[18]
*P*. *ostreatus*				
*P*. *ostreatus*	Wood chips, boll, sugar beet pellet pulp, palm fiber	Wheat bran, rice bran, soya cake powder, rice bran, carrot pulp	Highest yield for boll, beet pellet and palm fiber with supplements.	[19]
*P*. *ostreatus*	Diaper and food waste.	N.A.	Showed it could grow.	[20]
*P*. *ostreatus*	Acacia sawdust, corncob, sugarcane bagasse	N.A.	Highest growth on corncob and sugarcane bagasse.	[21]
*P*. *ostreatus*	Poplar sawdust.	Food waste compost, rice bran	Optimum growth rate for 12% rice bran and 25% food waste	[22]
*P*. *ostreatus*	Rice straw, wheat straw, paper, sugarcane bagasse, sawdust of alder	Rice bran	Rice straw alone was found to give the highest yield	[23]
*P*. *ostreatus*	Potato dextrose agar, yam dextrose agar, sweet potato dextrose agar, malt extract agar	Molasses, glucose	Potato and yam dextrose agar led to the highest yield. Largest colony growth with 1 to 5% sucrose supplement.	[24]
*P*. *ostreatus*	Wheat stalk, millet stalk, soybean stalk, cotton stalk	N.A.	Highest yield on soybean stalk.	[25]
*P*. *ostreatus*	Sawdust from *Triplochiton scleroxylon*, rice straw, banana leaves, maize, corn husk, rice husk, elephant grass	N.A.	Highest yield on sawdust. The yield was found to correlate with the high cellulose, lignin and fibre content of the substrate.	[26]
*P*. *ostreatus*	Alfalfa, barley hay, sawdust (not specified), wheat hay	Salts	Highest growth on wheat hay and distilled water.	[27]
*P*. *ostreatus*	Sawdust (not specified)	Cassava peel, yam peel, plantain leaf	Sawdust supplemented with 10% cassava peel led to the best results.	[28]
*P*. *ostreatus*	Wheat straw	Grape pomace	Some toxicity from the grape pomace.	[29]
*P*. *ostreatus*	Sawdust from beech, oak and poplar, wheat and rye straw, by-products from the textile industry, flax and hemp shives	N.A.	Wheat and rye straw with flax shives led to the highest yield.	[30]
*P*. *ostreatus*	Empty fruit bunch and sugarcane bagasse	N.A.	Empty fruit bunch can be used.	[31]
*P*. *ostreatus*	Wheat straw, cotton gin-trash, peanut shells, poplar, oak sawdust, corn cobs, olive press-cakes	N.A.	Highest growth on the cotton gin-trash, peanut shells and poplar sawdust.	[32]
*G*. *lucidum &*	Wheat straw	Olive mill waste and olive pruning residues	Small concentration in olive mill and pruning residues increased the protein content in both fungi. Generally, *P*. *ostreatus* grew more than *G*. *lucidum*	[12]
*P*. *ostreatus*				
*G*. *lucidum &*	Cellulose	Potato dextrose	Cellulose with potato dextrose led to higher growth presumably because simplest to digest.	[33]
*P*. *ostreatus*				
*P*. *ostreatus*	Cocopeat with sawdust (not specified)	N.A.	50% cocopeat led to the highest yield.	[34]

Employing supplement sources derived from the food industry is promising in the scope of a circular economy. Indeed, growth of mycelium for mycelium-derived products could be a means to utilize food waste which amounts to one third of the total food production per year [35] and 44% of the municipal solid waste, according to the World Bank 2018 [36]. Similar to other living organisms, fungi accumulates various minerals and nutrients, which in turn influence their morphology, growth and behaviour [37, 38]. Among the various food sources used as enriched and fortified nutrients for humans and animals, the most common ones are cereal crops and fatty seeds [39]. For example, wheat, rice, corn, barley, oats, or flaxseed are commonly used as supplements for dairy cows and other human and animal consumption [40, 41]. In general, the determination of whether a substrate and the accompanying supplements are suitable for mycelium growth is explored by investigating the mass of mycelium grown. Literature lacks data with regards to the morphology and composition of the mycelium formed, as well as of the supplements used. Furthermore, some supplements were found to exhibit better properties only in conjunction with selected substrates and only for specific fungus species.

In this paper, we complement current research on the effect of substrates composition and preparation by exploring the impact of commonly available food sources on the growth of *P*. *ostreatus* and *G*. *lucidum*. To this aim, experiments on the growth compatibility of mycelium with substrates from bamboo and wood chips and barley, oats, and flaxseeds were carried out and characterised using electron microscopy and elemental analysis. Indeed, composition and morphology of the mycelium are two key factors that significantly contribute to the properties of the final mycelium-bound composites [42, 43]. It was observed that the common crop barley is effective in boosting the growth of both *P*. *ostreatus* and *G*. *lucidum* in non-optimized substrate conditions. The additional nutrients brought by the supplements were able to grow a larger amount of mycelium as compared to the same substrate without the food. The results of this work are expected to contribute to enhancing the fabrication process of mycelium-based products for a large variety of solid waste-based substrates.

## 2. Materials and methods

### 2.1. Materials

*G*. *lucidum* and *P*. *Ostreatus* were sourced from Malaysian Feedmills Farms, Malaysia and Kin Yan, Singapore, respectively. *Dendrocalamus Asper* bamboo were collected from Indonesia and dried in an oven (IKA, Malaysia) at 80°C for one week. They were subsequently grinded with the Fritsch cutting mill pulverisette 15 and sieved by the Fritsch Vibratory Sieve Shaker Analysette 3 Spartan to obtain 200 μm-length fibres.

The food supplements were staple food purchased from the local supermarket including brown flaxseed meal (Origins, Healthfood, product of U.S.A., HACCP certified), organic pearl barley (Origins, Healthfood, product of U.S.A., HACCP certified), instant oatmeal (Quaker, 100% Australian wholegrain oats, product of Malaysia). The wood chips were purchased from Vadigran, Belgium. The flaxseeds were used as purchased, whereas the barley and oats were grounded with a blender mixer (PowerPac) for approximately 7 and 3 minutes, respectively. The wood chips were also ground for 7 minutes.

### 2.2. Characterisation of the food supplements and substrates

Small samples were removed from the bulk with tweezers and placed into a drying oven (IKA, Malaysia) overnight at 48°C. Electron micrographs were obtained using a scanning electron microscope (SEM) JSM-5510LV, JEOL, Japan on samples deposited onto a carbon tape and coated with 45 seconds of gold using the Cressington 108 Gold Sputter Coater, United Kingdom. The size distributions were obtained by measuring sizes from the micrographs using the software Image J (NIH, U.S.A.) and calculated from more than 100 measurements. The mineral content was determined from the packaging information. Fourier-transform infrared spectroscopy (FTIR) was conducted with a FTIR Spectrometer (Frontier, United States) using the Attenuated Total Reflectance method. Each composition was measured for at least 3 times on different samples and the average measurement was used for calculation purpose. The pH values of the substrates were measured using pH paper and was neutral between 6 and 7 for all substrates.

### 2.3. Growth of the mycelium

All tools and containers were sterilized with an autoclave (MaXterile 60, Daihan, South Korea) at 121°C for 45 minutes. The substrates inoculated with the fungus were deposited in a recipient, moisturized to maintain a humidity of nearly 99% and fresh air exchange was also ensured. The growth experiment was conducted up to 10 days for *G*. *lucidum* and for 27 days for *P*. *ostreatus*. For both fungi, the samples were kept in the laboratory environment, with a temperature of 22 ± 0.5°C. The compositions were varied following **Tables 2 and 3**. The weights included in the tables are the dry weights of the ingredients measured before the addition of water. The growth with the supplements were carried out for at least three times.

**Table 2:** Composition used in the growth experiments with the three food supplements and the two fungus species.

*Fungus species*	Lignocellulosic substrate	Amount of food supplement	Amount of inoculum	Amount of water
*G*. *lucidum*	1 g bamboo	0.5 g barley	0.5 g	3 g
*G*. *lucidum*	1 g bamboo	0.5 g oats	0.5 g	3 g
*G*. *lucidum*	1 g bamboo	0.5 g flaxseed	0.5 g	3 g
*P*. *ostreatus*	2 g bamboo	2 g barley	1 g	3 g
*P*. *ostreatus*	2 g bamboo	2 g oats	1 g	3 g
*P*. *ostreatus*	2 g bamboo	2 g flaxseed	1 g	3 g

**Table 3:** Composition used in the growth experiments with barley, and the two lignocellulosic substrates bamboo, and wood chips, at different mycelium to water ratios.

*Fungus species*	Lignocellulosic substrate	Amount of barley supplement	Amount of inoculum	Amount of water
*P*. *ostreatus*	2 g bamboo	1 g	0.5 g	3 g
*P*. *ostreatus*	2 g bamboo	1 g	1.5 g	3 g
*P*. *ostreatus*	2 g bamboo	1 g	2 g	3 g
*P*. *ostreatus*	2 g bamboo	1 g	2 g	6 g
*P*. *ostreatus*	2 g wood chips	1 g	0.5 g	3 g
*P*. *ostreatus*	2 g wood chips	1 g	1.5 g	3 g
*P*. *ostreatus*	2 g wood chips	1 g	2 g	3 g
*P*. *ostreatus*	2 g wood chips	1 g	2 g	6 g

### 2.4. Characterisation of the mycelium

At each specific time point during the growth, the containers were opened, and a sample of mycelium was taken out. The earliest day for sampling was day 7 due to the lack of mycelium optically visible at earlier days. Small samples were dried overnight at 48°C before being sputtered with gold for 45 s and observed in SEM. Lengths of hyphae and pore diameters were measured more than 100 times using the software Image J. FTIR was conducted using a FTIR Spectrometer and the Attenuated Total Reflectance method. Each composition was measured at least 3 times on different samples and the average measurement was used for calculation purpose.

## 3. Results and discussion

### 3.1. Effects of food supplements on the growth of *G*. *lucidum* and *P*. *ostreatus*

**Fig 2:**
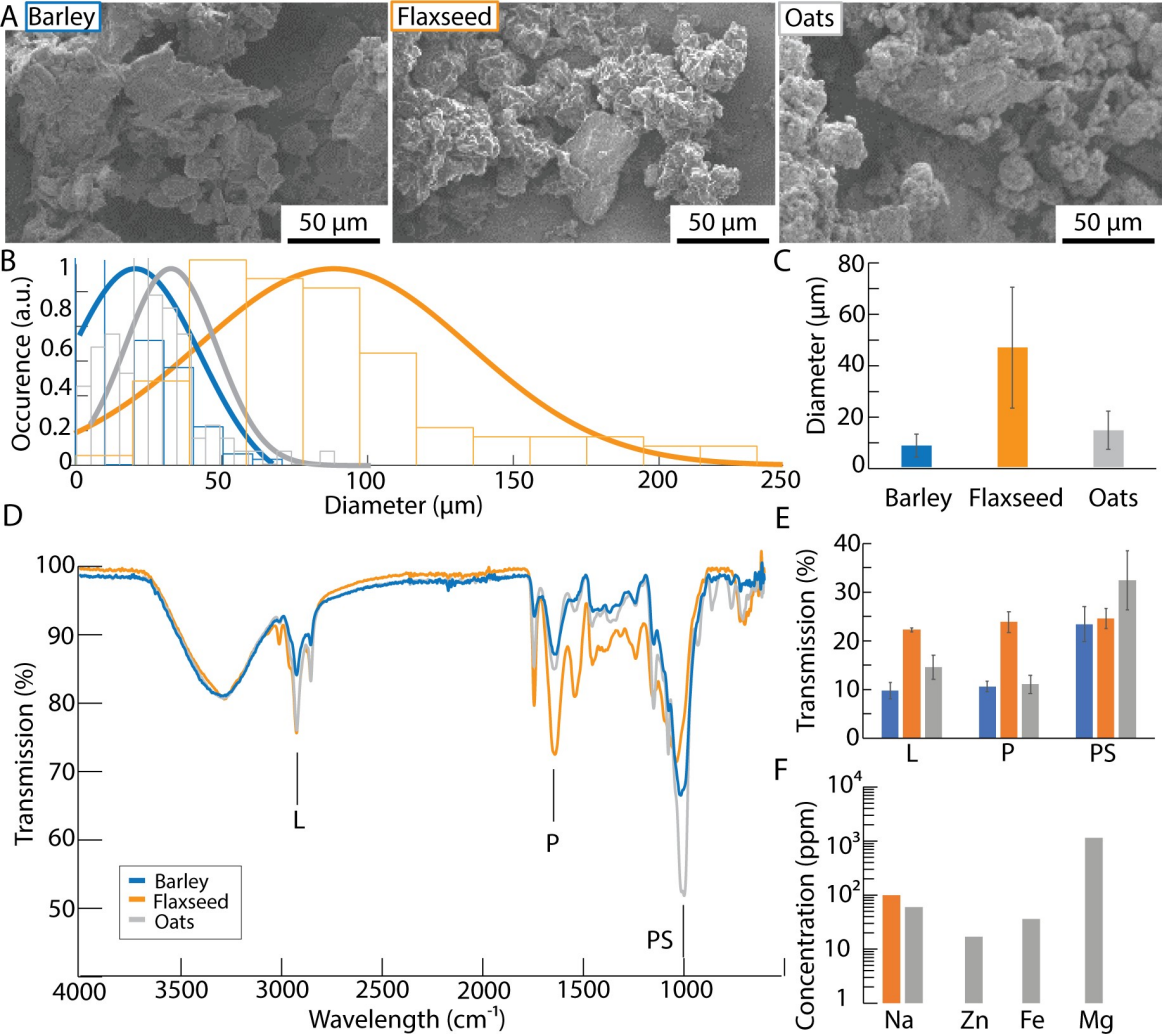
**(A)** Electron micrographs of grounded barley (blue), flaxseed (orange) and oats (grey). Size distribution **(B)** and average diameter and standard deviation **(C)** for each food supplement. **(D)** Representative FTIR spectra. L indicates lipides, P proteins, and PS polysaccharides. **(E)** Transmission intensity corresponding to the content in lipides, proteins, polysaccharides obtained from the FTIR. **(F)** Mineral concentration in each supplement.

Three sources for supplement, including pearl barley, flaxseed and oats were selected for this study. These foods are commonly found in households across the world. Prior to using them to grow mycelium, they were characterized in terms of size and nutritious content. The size distribution of each food supplement was of an average of 20 ± 9 μm for barley, 87 ± 47 μm for flaxseed, and 35 ± 15 μm for oats (**Fig 2A–2C**). The hardness of the flaxseeds prevented their grinding to smaller sizes as compared to barley and oats. However, the size distribution for each food supplement was in the range of 10–100 μm. The compositions of the food supplements were obtained using FTIR and the nutritional values were obtained from the food packaging. FTIR is a method commonly used for semi-quantitative analysis [44, 45]. We used FTIR to be able to directly compare the components of the food supplements in the three categories: lipids, proteins, and polysaccharides. The FTIR measurements were validated by the information provided on the packaging of the foods. Typically, the frequencies at 3200–2800 cm^-1^ are attributed to the stretching of CH_2_ and CH_3_ found in lipids. The vibration bands at 1560–1525 cm^-1^ are attributed to the bending of N-H and C-H in proteins. The vibrations at 1200–900 cm^-1^ are attributed to the stretching of C-O, C-C, P = O and C-O-C of polysaccharides. The broad absorption band at 3500–3000 cm^-1^ are representing vibrations of water molecules. The FTIR spectra of the food supplements showed different lipids, protein and polysaccharide contents (**Fig 2D**). The results indicate that oats had the highest content of polysaccharides, whereas flaxseeds had the highest content of lipids and proteins. This trend follows the nutritional indications from the food packaging. Furthermore, various minerals were also found to be part of the food sources, especially for the oats which were fortified with Fe, Zn, and Mg.

**Fig 3:**
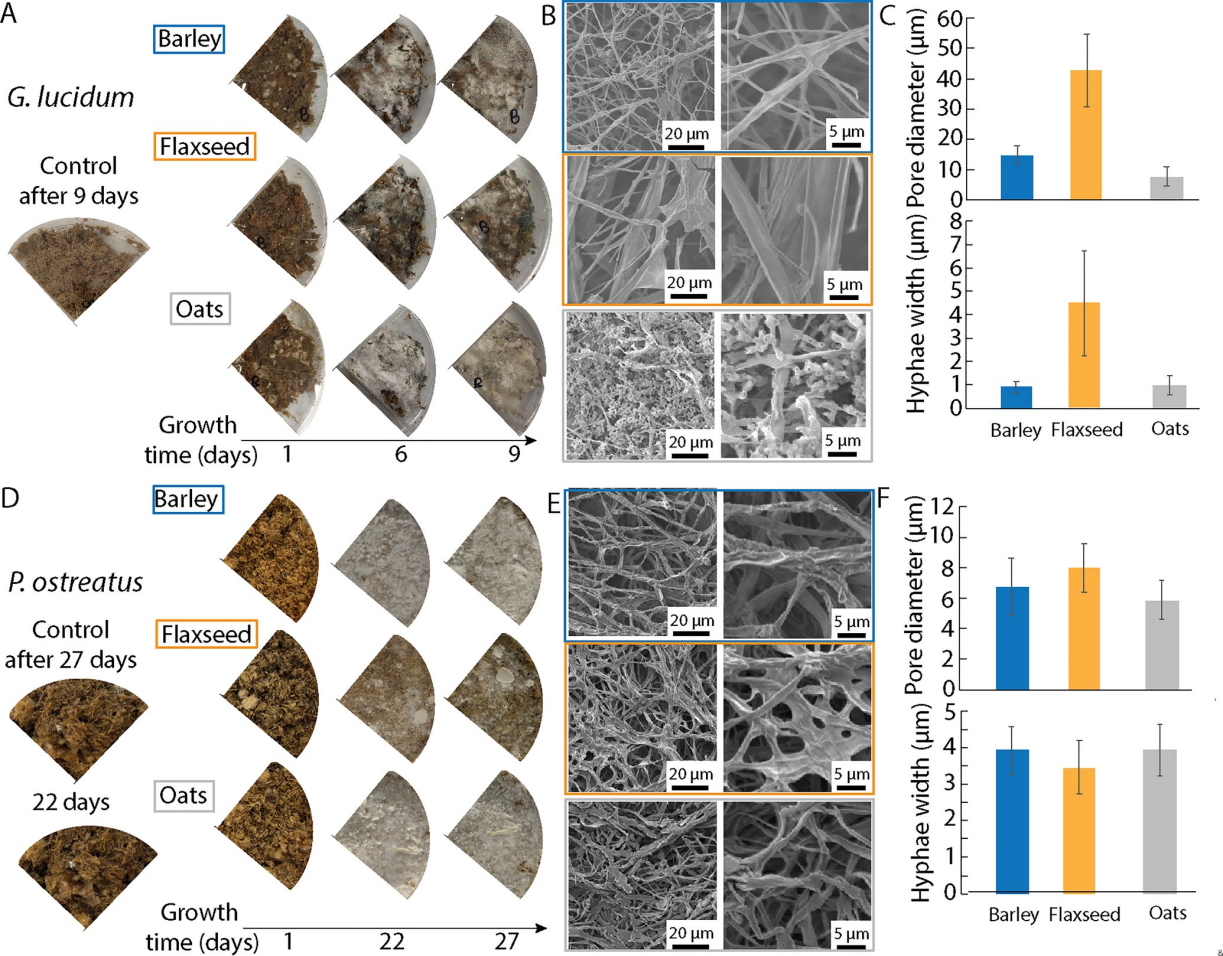
Effects of nutritious supplements on the growth of *G*. *lucidum* **(A-C)** and *P*. *ostreatus* **(D-F)** on bamboo substrate. **(A)** Pictures showing the growth of *G*. *lucidum* at 1, 6 and 9 days for the three food supplements and control sample without food. **(B)** Electron microscope images showing the mycelium after 9 days growth. **(C)** Pore diameter and hyphae width of the grown mycelium after 9 days, respectively. **(D)** Optical images showing the growth of *P*. *ostreatus* at 1, 22 and 27 days. **(E)** Electron microscope images showing the mycelium after 27 days growth. **(F)** Pore diameter and hyphae width of the grown mycelium after 9 days, respectively. Blue corresponds to barley supplement, orange to flaxseed and grey to oats.

**Fig 4:**
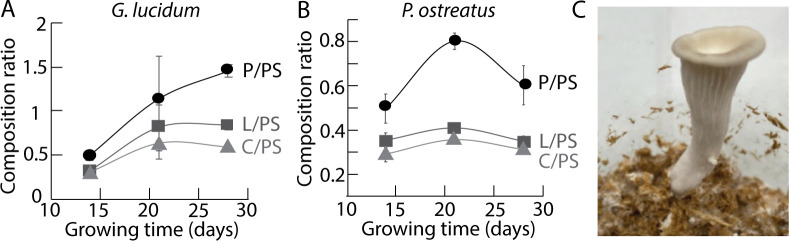
Composition ratios as a function of the growing time for *G*. *lucidum* **(A)** and *P*. *ostreatus* **(B).** P stands for protein, PS polysaccharide, L lipid and C chitin. For each fungus, the data for each time point have been averaged over all the experiments carried out with food supplements, irrespective of the nature of the foods. **(C)** Picture of a fruiting body of *P*. *ostreatus* that developed onto the substrate after 20 days growth.

The effects of these food supplements on the growth of *G*. *lucidum* and *P*. *ostreatus* were studied using bamboo fibers as the substrate (**Figs 3 and 4**). Without the nutritious supplements, no growth could be found, probably due to a non-optimal combination of the other parameters, namely temperature, pH, concentration, humidity, light, etc. Nevertheless, in the presence of the supplements, all substrates exhibited growth which was visualized directly by white areas on and inside the substrate, denoting the presence of mycelial runs.

*G*. *lucidum* grew extensive mycelium after only one week, in presence of barley and oats (**Fig 3A**). The mycelium grown also exhibited different morphologies depending on the supplement, which were investigated using scanning electron microscopy (**Fig 3B**). The hyphae, *i*.*e*., the mycelium filaments, grown on barley and flaxseed-enriched substrates appeared straight, whereas round tree-like and hollow tube structures were found on the mycelium grown on oats-enriched substrates. Such highly-interconnected structures have been previously reported in presence of chitosan, cellulose, or potato-dextrose [33, 46, 47]. Furthermore, the interconnected network appeared more porous and with wider hypha for the flaxseed-enriched substrate as compared to the other two (**Fig 3C**). For the enhanced growth of healthy mycelium, barley as a nutritious supplement is therefore preferrable. The structure and porosity of the mycelium are important for the properties of the resulting composite, for example hydrophobicity and mechanical properties [33]. A tighter mycelium network is therefore likely to yield a mycelium-bound biocomposite with higher mechanical properties.

The effects of the three food supplements on *P*. *ostreatus* were also investigated. The experiments were conducted over a longer period of time as compared to *G*. *lucidum*, due to the slower mycelium growth of *P*. *ostreatus*, which is likely due to a non-optimal combination of the overall parameters, namely concentration of the substrate, food and inoculum, humidity, light, etc. (**Fig 3D**). The barley and oats-enriched substrates exhibited a homogeneous and extensive mycelium growth that was highly visible after 22 and 27 days. However, there was heterogeneous growth on the flaxseed-enriched substrates with a few millimetric patches of dense and compact mycelium. The pore diameters and the hyphae widths did not show any significant difference between the substrates, and the tube-like features observed with *G*. *lucidum* were not found in these samples (**Fig 3E, 3F**). However, some areas in the mycelium grown on the flaxseed-enriched substrate did also exhibit large hyphae width (**Fig 3E**).

Barley as a food supplement appeared to be suitable for the development of healthy mycelium for both *G*. *lucidum* and *P*. *ostreatus*, without requirement of a careful optimization of the growth conditions. Since the efficacy of degrading enzymes increases with the increase of surface area [48], the smaller size and larger surface area of the barley supplement as compared to the other two supplements was likely favourable. Flaxseeds were found not to be efficient for mycelium growth, which could also be linked to their larger size and their lower content in polysaccharides. Also, the high lipid content might have induced changes in the mycelium morphology due to a rise in hydrophobicity. Oats were suitable for *P*. *ostreatus* but the morphology of the mycelium of *G*. *lucidum* was found to be unusual with hollow tube shapes, which might lead to inhomogeneities and weaker mechanical properties. To increase the yield of mycelium growth on substrates made of bamboo microfibers, the addition of barley as a food supplement is therefore promising.

### 3.2. Comparison of the growth of *G*. *lucidum* and *P*. *ostreatus* on bamboo fibers with food supplements

To further characterize the effects of food supplements on the mycelium growth, FTIR was carried out as a function of time. Indeed, as the fungus is degrading the substrate, it builds a dense mycelium network composed of lipids, protein, chitin, and polysaccharides. Lipids, proteins and polysaccharides can be deduced from the vibrations as described above for the characterization of the substrate. Chitin is an additional band around 1375–1365 cm^-1^ for the bending of C-H. Based on the FTIR spectra, composition ratios were calculated. These are the ratios between the peak intensities for proteins (P), lipids (L) and chitin (C), respectively, with the peak intensity for polysaccharides (PS). No major variability between the composition of the mycelia grown with the different food supplement could be recorded. However, a general trend could be seen, that was essentially species-dependent (**Fig 4A and 4B**). The growth of *G*. *lucidum* was indeed continuously increasing with time, whereas *P*. *ostreatus* grew quickly during the first two weeks, then the growth rate dropped. This decrease in growth is likely due to the fruiting of the fungus after 20 days (**Fig 4C**). This result indicates that food supplements cannot overcome the differences between fungal species and modify significantly the chemical composition of the mycelium. In the context of mycelium-bound composites, the characteristics of the fungal strain would therefore determinate the mycelium growth kinetics and chemical composition, whatever the food supplement used. However, for one selected fungal strain, food supplements can boost the production of mycelium and induce some modification in its morphology.

### 3.3. Effect of barley supplement on the growth of *P*. *ostreatus* on different substrates

**Fig 5:**
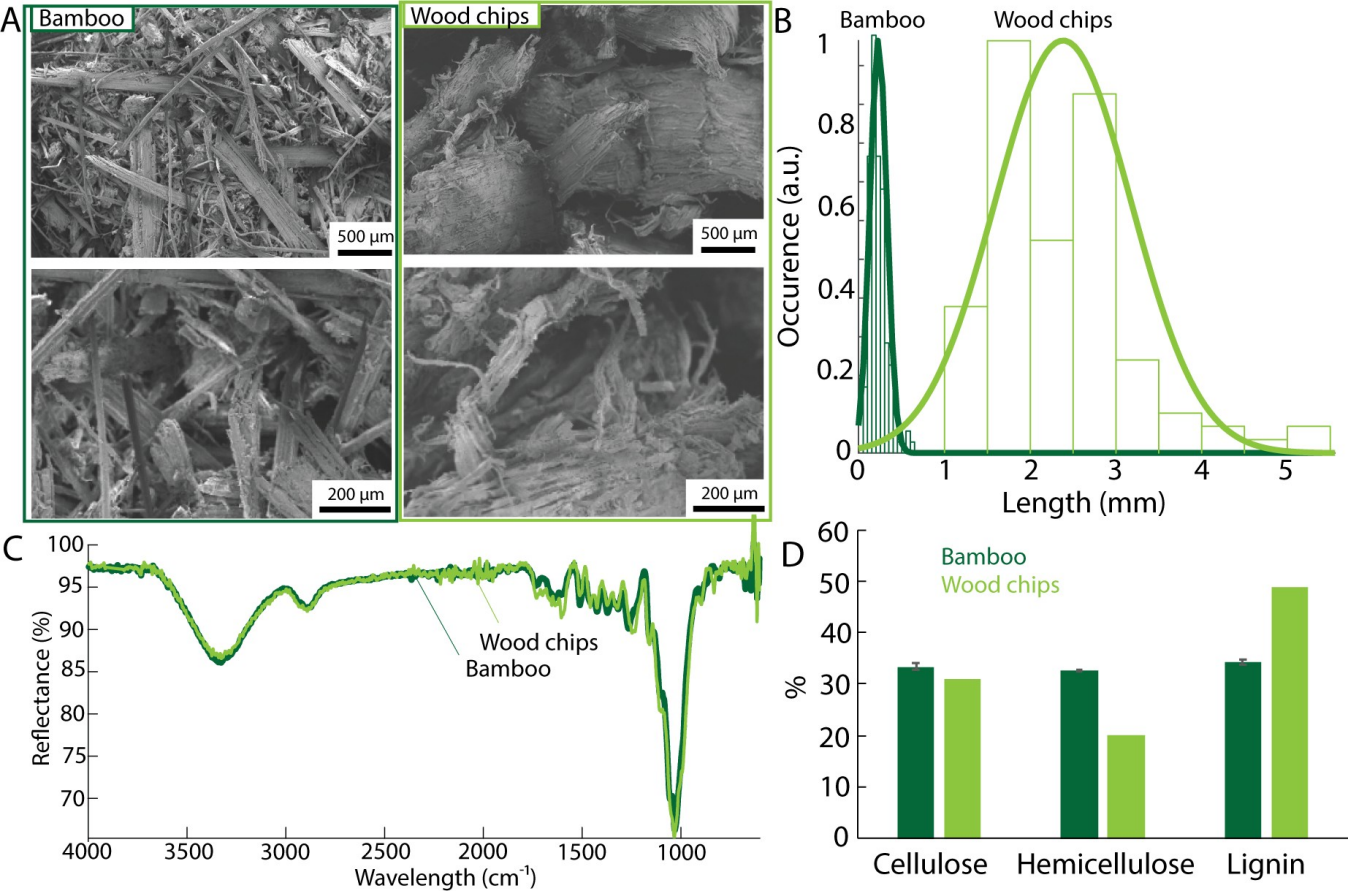
**(A)** Electron micrographs of the bamboo fibers and wood chips used as substrate. **(B)** Size distribution of the bamboo fibers and the wood chips. **(C)** Representative FTIR spectra. **(D)** Cellulose, hemicellulose and lignin content in % in the substrates. Data estimated from [49–51].

To test that barley enrichment can be used to increase mycelium growth on lignocellulosic substrates without requiring careful optimization of growth conditions, a commercial wood chips blend was obtained from a pet store as a substrate. We demonstrate here the advantage of using barley as a food supplement to boost the growth of *P*. *ostreatus*. As compared to the bamboo substrate, the wood chips had larger dimensions but no major difference in the FTIR spectra could be recorded between the bamboo fibers and the wood chips (**Fig 5**). However, it is commonly reported that wood has a higher lignin and lower hemicellulose content as bamboo [49–51]. The similarity between the FTIR spectra of bamboo and wood chips is likely related to the pre-processing of the bamboo microfibers, involving grinding, shredding and the heat treatment at 80°C.

**Fig 6:**
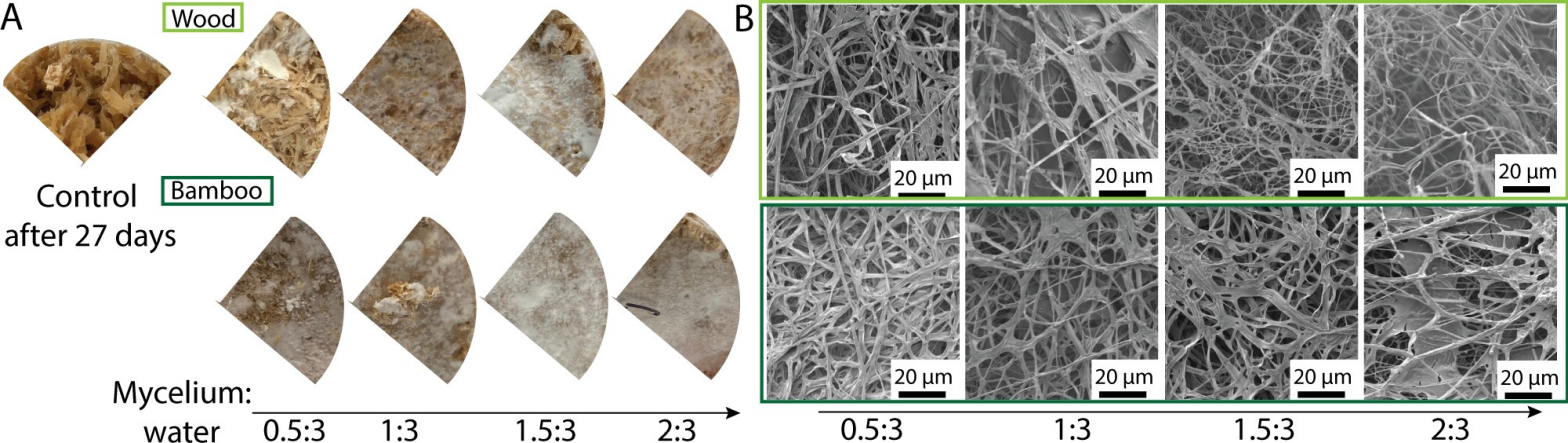
Effects of barley supplement on the growth of *P*. *ostreatus* on bamboo and wood substrates. **(A)** Optical images of *P*. *ostreatus* mycelium grown after 27 days on each lignocellulosic substrate, in presence of barley supplement, for different mycelium to water ratios. The image on the left is the control sample after 27 days of growth on wood substrate, without supplement. **(B)** Electron micrographs of the corresponding mycelium. Light green are wood chips substrates and dark green are bamboo substrates.

Without barley as nutritious supplement, the wood chips did not lead to the growth of mycelium from *P*. *ostreatus* after 27 days (**Fig 6A**). This was predicted since large dimensions and increased lignin content are generally not favourable for mycelium growth. However, in the presence of barley supplement at 1 g for 2 g of mycelium spawn, significant growth was observed at varying ratios of mycelium to water content. In this set of experiments, the substrate and food contents were maintained constant while the mycelium and water content varied. For both bamboo and wood substrates, higher water content was more favourable for the growth of the mycelium, probably due to the water absorption of the pure substrate. A mycelium to water content of 1.5 to 3 seemed to be the best ratio for mycelium growth. No significant variations in the pore diameter and hyphae width were recorded between the samples after 27 days of growth (**Fig 6B**). These results indicate that adding barley as a food supplement is indeed a simple means to increase the mycelium growth, even on unfavourable substrates. It is likely that the mycelium could develop by primarily degrading the barley rather than degrading the substrate. However, this hypothesis is difficult to verify as the elements of the composite, namely mycelium, wood chips, and food supplement, are difficult to separate. Furthermore, the system is synergetic: in absence of wood substrate and in presence of the food supplement, the mycelium cannot grow properly and contamination by other species occurs quickly. It could also be hypothesized that the food supplement increases the energy of the fungus which could then synthesize more enzyme to degrade the substrate as well as increase its resistance against contamination. Following the secretion of enzymes with time in the presence of foods could help answer these questions.

## 4. Conclusions

In summary, the effects of the addition of food supplements on the growth of mycelium of two fungal species, *G*. *lucidum* and *P*. *ostreatus* were studied. Between flaxseed, oats, and barley, it appeared that barley led to significant mycelium growth for both fungi on bamboo and wood chips substrates. What is particularly interesting is the absence of mycelium growth without the food supplement, indicating a significant effect of this simple strategy on mycelium growth. Furthermore, food supplements rich in carbohydrates, such as barley, provided a more significant increase in growth as compared to those rich in lipids. Also, smaller size was favourable, probably by facilitating the action of degrading enzymes from the mycelium.

Our results therefore suggest that bringing additional nutrients derived from common food is an interesting strategy to grow mycelium-bound composites on various substrates, without the need for careful optimization of the growth. This approach can not only valorize food waste and green waste into useful materials, but can also be potentially implemented by local manufacturers without the need for intensive training and specialisation. Indeed, we have shown that the addition of barley could increase the mycelium growth for two different fungal species, despite the growth conditions being not optimized in terms of temperature, moisture, inoculum to substrate ratio. The approach could likely be effective for other fungal species as well and be used as a simple standard method to produce mycelium-bound composites on various substrates. It could also be envisaged to use the "energy boost" from the food supplements to help the mycelium degrade tougher substrates, including plastics.

Overall, mycelium-bound composites have the potential to develop local circular economy. The results from this study are expected to contribute to the studies of mycelium-bound materials and their applications.

## Supporting information

S1 Graphical abstract (TIF)
