# Supplementary figures and images for: Effect of common foods as supplements for the mycelium growth of Ganoderma lucidum and Pleurotus ostreatus on solid substrates
Source: PLoS One. 2021 Nov 30;16(11):e0260170. doi: 10.1371/journal.pone.0260170 (PMC8631619; doi:10.1371/journal.pone.0260170)

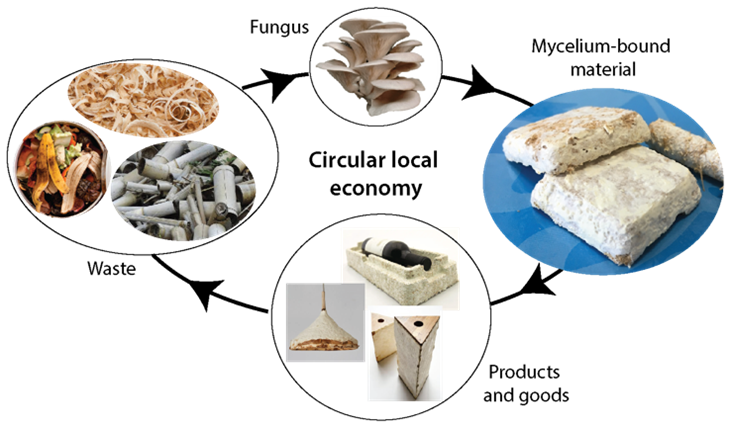

Supplement: S1 Graphical abstract — (TIF) [file pone.0260170.s001.tif]
